# Reference ranges of the WHO Disability Assessment Schedule (WHODAS 2.0) score and diagnostic validity of its 12‐item version in identifying altered functioning in healthy postpartum women

**DOI:** 10.1002/ijgo.12466

**Published:** 2018-05-23

**Authors:** Jussara Mayrink, Renato T. Souza, Carla Silveira, José P. Guida, Maria L. Costa, Mary A. Parpinelli, Rodolfo C. Pacagnella, Elton C. Ferreira, Maria H. Sousa, Lale Say, Doris Chou, Veronique Filippi, Maria Barreix, Kelli Barbour, Peter von Dadelszen, José G. Cecatti, the Brazilian Cohort on Severe Maternal Morbidity (COMMAG) study group and the WHO Maternal Morbidity Working Group (MMWG), Carla B. Andreucci, Carina R. Angelini, Juliana P. Ferraz, Dulce M. Zanardi, Rodrigo S. Camargo, Sara Cottler, Olubukola Fawole, Tabassum Firoz, Luis Gadama, Atf Ghérissi, Gill Gyte, Michelle Hindin, Anoma Jayathilaka, Amanda Kalamar, Yacouba Kone, Isabelle Lange, Laura A. Magee, Arvind Mathur, Affette McCaw‐Binns, Mark Morgan, Stephen Munjanja, Gathari N. Gichuhi, Max Petzold, Elizabeth Sullivan, Frank Taulo, Özge Tunçalp, Rachel Vanderkruik

**Affiliations:** ^1^ Department of Obstetrics and Gynecology University of Campinas São Paulo Brazil; ^2^ UNDP–UNFPA–UNICEF–WHO–World Bank Special Programme of Research Development and Research Training in Human Reproduction (HRP) Department of Reproductive Health and Research WHO Geneva Switzerland; ^3^ Department of Infectious Disease Epidemiology London School of Hygiene and Tropical Medicine London UK; ^4^ Department of Obstetrics and Gynecology University of Utah Salt Lake City UT USA; ^5^ Molecular and Clinical Sciences Research Institute St George's University of London London UK

**Keywords:** Postpartum period, Puerperium, Severe maternal morbidity, WHODAS‐12, WHODAS‐36

## Abstract

**Objectives:**

To compare scores on the 36‐item WHO Disability Assessment Schedule 2.0 tool (WHODAS‐36) for postpartum women across a continuum of morbidity and to validate the 12‐item version (WHODAS‐12).

**Methods:**

This is a secondary analysis of the Brazilian retrospective cohort study on long‐term repercussions of severe maternal morbidity. We determined mean, median, and percentile values for WHODAS‐36 total score and for each domain, and percentile values for WHODAS‐12 total score in postpartum women divided into three groups: “no,” “nonsevere,” and “severe” morbidities.

**Results:**

The WHODAS‐36 mean total scores were 11.58, 18.31, and 19.19, respectively for no, nonsevere, and severe morbidity. There was a dose‐dependent effect on scores for each domain of WHODAS‐36 according to the presence and severity of morbidity. The diagnostic validity of WHODAS‐12 was determined by comparing it with WHODAS‐36 as a “gold standard.” The best cut‐off point for diagnosing dysfunctionality was the 95th percentile.

**Conclusion:**

The upward trend of WHODAS‐36 total mean value scores of women with no morbidity compared with those with morbidity along a severity continuum may reflect the impact of morbidity on postpartum functioning.

## Abstract 2

WHODAS total mean scores among postpartum women showed an upward trend in women with no morbidity compared with those with morbidity along a severity continuum.

## 1 INTRODUCTION

Progress in maternal health and the consequent reduction in maternal mortality are considered important goals worldwide, as part of the fifth Millennium Development Goal and, presently, of the third Sustainable Development Goal.^1^, ^2^ Since the 1990s, there has been significant improvement in maternal and perinatal health indicators, with a decline of about 50% in the overall maternal mortality rate.^3^ Improvements in antenatal care,^4^ as well as access to institutional deliveries, using clean delivery kits,^5^ and interventions to prevent and manage hypertensive disorders and postpartum hemorrhage^6^, ^7^ have likely contributed to that reduction. However, there are still many challenges to ascertain women's health and well‐being during pregnancy and the postpartum period, even among those women with no medical complications, especially in low‐ and middle‐income countries.

The postpartum period is characterized by multiple concerns involving self‐confidence, mother–infant interaction, body image experiences, adjustment to maternal roles, and attitudes.^8^ These concerns—which are not only dependent on the presence or diagnosis of morbidity, but also on the inadequate assessment of such issues or even their misinterpretation—may contribute to the deterioration of maternal and newborn health.^9^

The occurrence of severe maternal morbidity (SMM), defined as having a potentially life‐threatening condition (PLTC) and/or maternal near miss (MNM), has been studied over the past decade: impacts on maternal and child health are unquestionable,^10^, ^11^, ^12^ as are their effects on women's functionality (her ability to perform everyday tasks, including social and economic responsibilities).^13^ Non‐life‐threatening or nonsevere maternal morbidity (non‐SMM) is also a theme of concern and is currently defined as “any health condition attributed to and/or complicating pregnancy and childbirth that has a negative impact on the woman's well‐being and/or functioning”.^3^ However, even among new mothers without morbidity, the inherent complexity of pregnancy and the postpartum period should be considered when providing care to this specific population.

Monitoring more than just the traditional indicators of health, pregnancy, and childbirth requires an expanded approach. Information on disability and functioning is an important component of health assessment and has provided helpful evidence for measuring disease burden across different settings,^14^, ^15^ through a tool developed by the WHO called the WHO Disability Assessment Schedule 2.0 (WHODAS 2.0).^16^ This tool seeks to measure functionality, and considers six domains (cognition, mobility, self‐care, getting along with people, life activities, and participation) as they apply to daily living activities in the 30 days preceding the tool's application. The complete version has 36 questions (WHODAS‐36), while a shorter 12‐item version is also available (WHODAS‐12). However, the WHODAS has not often been applied to women of reproductive age, during pregnancy, or the postpartum period.

To better understand WHODAS scores, and their distribution, among postpartum women, with the intention of suggesting a cut‐off point for screening, we applied this instrument to women with no morbidity and those on a continuum of morbidity (any morbidity to SMM). We further validated the shorter WHODAS‐12 using the full 36‐item version as a reference for the assessment of disability and functioning in nonmorbid postpartum women.

## 2 MATERIALS AND METHODS

This is a secondary analysis of the Brazilian Cohort on Severe Maternal Morbidity (COMMAG)—a retrospective cohort study that included women who delivered between July 1, 2008, and June 30, 2012, at the Women's Hospital of the University of Campinas, Brazil. The study was a long‐term evaluation of the consequences of PLTC and MNM incidents using a multidimensional approach.^17^ The details of the methods used for the main study and primary results on WHODAS have been published elsewhere.^13^, ^18^ Briefly, cases of SMM were identified using the standardized WHO criteria,^19^ as PLTC and MNM, and compared with a randomly selected control group, without SMM. One of the instruments used to assess women's functioning status was the WHODAS 2.0 (for both cases and controls). Participants provided informed consent and the University of Campinas institutional review board provided approval for the original study.

For the current analysis, we aimed to select, from among the control group, all cases with no morbidity at all (during gestation, delivery, or postpartum). This involved applying the broad WHO definition for and criteria of maternal morbidity.^3^ Cases with any previous medical condition (hypertension, diabetes, anemia, cardiovascular disease, autoimmune disease, smoking, etc.) and cases with any complications documented during any pregnancy (pre‐eclampsia, hemorrhage, and infection, among others) were excluded from the no morbidity group. It is relevant to consider that our study included evaluations/interviews with women any time from 1 to 5 years postpartum and participants could have had other pregnancies during the intervening period. We hypothesized that any complication could impact the woman's WHODAS score. Considering this approach, we were able to identify three groups: SMM, non‐SMM, and no morbidity. We performed our analyses using these three groups. We used a virtual database built for the main study using the LimeSurvey platform (http://www.limesurvey.org; LimeSurvey GmbH, Hamburg, Germany).

Our group translated the instrument into Brazilian Portuguese.^13^ The total score for WHODAS ranges from 0–100, where a high score indicates major living limitations.^16^ The shorter WHODAS‐12 consists of two questions from each domain (called sentinel key questions) of the full 36‐item version of the WHODAS.^16^ A trained interviewer administered the WHODAS‐36 questionnaire (which only includes 32 questions if the participant is unemployed and no longer in school).

Sociodemographic, obstetric, and perinatal characteristics were described for each group considered. We determined mean, median, and percentile values for WHODAS‐36 total score, and separately for each domain. For each domain we excluded cases with missing data in any question. We also determined percentile values for WHODAS‐12 total score. For the short version, the detailed analysis on domains is not possible.^16^ The results were compared using the Kruskal‐Wallis test between groups. A *P* value of 0.05 or below was considered statistically significant.

We further validated the WHODAS‐12 (using the WHODAS‐36 as the “gold standard” for diagnosing disability and measuring functioning) for three percentiles: P90, P95, and P97.5. Finally, the best cut‐off points for screening accuracy of impaired functioning for both WHODAS‐36 and WHODAS‐12 were determined using receiver operating characteristic (ROC) curves for each endpoint. Our goal was to identify, from our sample, a group of women with no morbidity and to study their baseline WHODAS results in comparison with those of women presenting with maternal morbidity.

## 3 RESULTS

**Figure 1:**
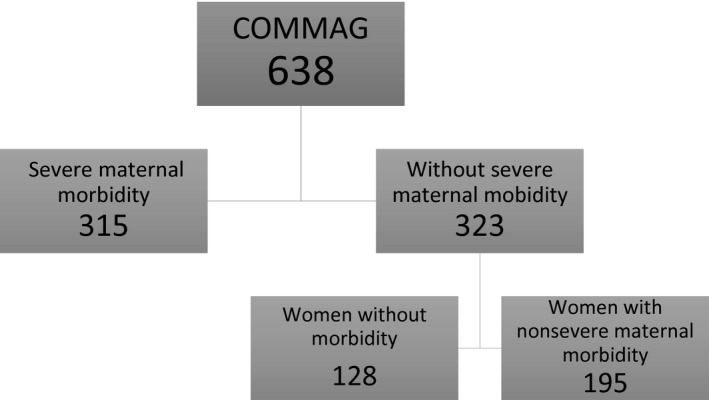
Flow chart of women included in the study.

Overall, there were 128 women with no morbidity (Fig. 1). Table 1 presents the sociodemographic and obstetric characteristics of these women as well as their perinatal outcomes. Women were generally young (mean age, 28.0 ± 6.4 years), multiparous, non‐white, and with a partner. The majority of deliveries were vaginal and nonoperative (57%), with low rates of prematurity and neonatal deaths.

**Table 1:** Sociodemographic and obstetric characteristics of women with no maternal morbidity (n=128)

Characteristics	No. (%)
Age, y	28.0 ± 6.4
<20	11 (8.6)
20–29	70 (54.7)
30–39	39 (30.5)
≥40	8 (6.3)
Parity	
1	58 (45.3)
≥2	70 (54.7)
Ethnicity	
White	57 (44.5)
Non‐white	71 (55.5)
Schooling	
<8 years	35 (27.3)
≥8 years	92 (72.7)
Relationship status	
No partner	22 (17.2)
Partner	106 (82.8)
Mode of delivery	
Vaginal	73 (57.0)
Operative vaginal delivery	10 (7.8)
Cesarean	45 (35.2)
Prematurity	
Yes	11 (8.6)
No	117 (91.4)
Perinatal outcome	
Alive	128 (100.0)
Neonatal outcome	
Discharged healthy	124 (96.9)
Early neonatal death (<1 week)	1 (0.8)
Late neonatal death (7–28 days)	1 (0.8)
Missing data	2 (1.6)

**Figure 2:**
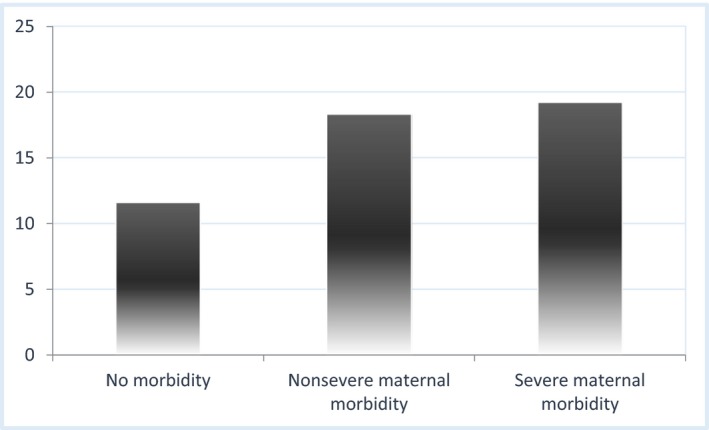
WHODAS‐36 mean total score in a continuum of severity, considering three groups: no morbidity, nonsevere maternal morbidity, and severe maternal morbidity. Kruskal‐Wallis test (*P*<0.05). [Colour figure can be viewed at http://www.wileyonlinelibrary.com

Considering the three groups of women on a continuum of severity, the WHODAS‐36 total mean scores were 11.58, 18.31, and 19.19 for no morbidity, non‐SMM, and SMM, respectively (Fig. 2). Table 2 shows mean and median values for each of the six WHODAS‐36 domains by morbidity category. For the no morbidity group, domains 1 (cognition) and 4 (getting along with people) presented the highest mean values. Scores for five domains of WHODAS‐36 (all except domain 4) were significantly different, generally depicting a dose‐dependent effect according to the presence and severity of morbidity (except for domains 1 and 4). The mean WHODAS scores were higher for women with ongoing medical diseases in comparison with those without.^20^ Additionally, we examined the 31 perinatal deaths in the sample (29 in the group of maternal morbidity), and found no significant impact of perinatal death on general or domain scores.

**Table 2:** WHODAS‐36 total score and specific domain values presented as mean, median, and standard deviation, for the three groups, no morbidity, nonsevere maternal morbidity, and severe maternal morbidity

Statistics	Women with no morbidity			Women with non‐SMM			Women with SMM			*P* value^a^
	Mean^b^	Median^b^	SD	Mean^b^	Median^b^	SD	Mean^b^	Median^b^	SD	
Total score	11.6	7.6	11.6	18.3	14.1	15.6	19.2	15.1	16.5	<0.001
Domain 1^c^	17.9	15.0	16.3	23.8	20.0	18.8	21.4	20.0	18.3	0.022
Domain 2	8.1	0.0	13.4	13.9	6.3	19.4	16.0	6.3	20.2	<0.001
Domain 3	2.4	0.0	6.3	5.9	0.0	13.1	6.2	0.0	13.5	0.028
Domain 4	13.9	8.3	20.4	14.9	8.3	19.6	14.6	8.3	20.6	0.478
Domain 5	11.8	0.0	18.1	22.8	10.0	26.7	26.1	12.5	28.8	<0.001
Domain 6	10.8	4.2	14.7	21.5	16.7	20.6	23.5	20.8	21.7	<0.001

Abbreviations: SD, standard deviation; non‐SMM, nonsevere maternal morbidity; SMM, severe maternal morbidity.

a Kruskal‐Wallis test (*P*<0.05).

b Range values of mean/median: 0–100.

c Domain 1: cognition (assesses communication and thinking activities); Domain 2: mobility (assesses activities such as standing, moving around inside the home, getting out of the home, and walking a long distance); Domain 3: self‐care (assesses bathing, dressing, eating, and staying alone); Domain 4: getting along with people (assesses interactions with other people and difficulties found due to a health condition); Domain 5: life activities (assesses difficulties in activities of daily life, associated with household chores, leisure time, work, and school); Domain 6: participation (assesses social dimensions, such as community activities, barriers and hindrances in the world around individual, and other problems such as personal dignity maintenance).

To evaluate the performance of the 12‐item version of the instrument, we compared WHODAS‐36 and WHODAS‐12 total scores for the nonmorbid group. Results were very similar (Table 3) when analyzed for different percentiles. For the complete WHODAS‐36, we were able to further analyze scores by domain: women scored highest (i.e. had a reduced functioning status) in domains 1 (cognition), 4 (getting along with people), and 5 (life activities).

**Table 3:** Values of percentiles 90, 95, and 97.5 for WHODAS‐36 total and domain scores, and for WHODAS‐12 total scores, among women with no morbidity (n=127^a^)

Percentiles by WHODAS‐36 domains and total scores (for WHODAS‐36 and ‐12)	Score (95% CI)
Domain 1: Cognition	
Percentile 90	45.0 (40.0–50.0)
Percentile 95	50.0 (45.0–57.8)
Percentile 97.5	55.0 (50.0–65.0)
Domain 2: Mobility	
Percentile 90	31.3 (18.8–43.8)
Percentile 95	43.8 (31.3–47.2)
Percentile 97.5	43.8 (43.8–56.3)
Domain 3: Self‐care	
Percentile 90	10.0 (10.0–13.2)
Percentile 95	17.0 (10.0–25.6)
Percentile 97.5	20.0 (11.2–40.0)
Domain 4: Getting along with people	
Percentile 90	50.0 (33.3–58.3)
Percentile 95	58.3 (50.0–83.3)
Percentile 97.5	81.5 (58.3–83.3)
Domain 5: Life activities	
Percentile 90	40.0 (30.0–50.0)
Percentile 95	50.0 (40.0–66.9)
Percentile 97.5	62.0 (47.5–83.3)
Domain 6: Participation	
Percentile 90	29.6 (25.0–42.8)
Percentile 95	44.5 (32.7–58.5)
Percentile 97.5	53.2 (42.1–75.0)
Total score (WHODAS‐36)	
Percentile 90	30.2 (21.7–38.0)
Percentile 95	38.7 (30.4–48.2)
Percentile 97.5	46.3 (35.5–50.9)
Total score (WHODAS‐12)	
Percentile 90	30.9 (23.3–36.1)
Percentile 95	37.4 (32.3–44.1)
Percentile 97.5	43.3 (35.6–50.0)

a One woman was excluded from this analysis because there was one missing response for Domain 5.

**Table 4:** Diagnostic validity of WHODAS‐12 using WHODAS‐36 as a “gold standard” to determine dysfunction and disability among women in the postpartum period

	P>90	P>95	P>97.5
Sensitivity	83.3%	83.3%	66.7%
Specificity	98.3%	99.2%	99.2%
Positive predictive value	83.3%	83.3%	66.67%
Negative predictive value	98.3%	99.2%	99.2%
AUC ROC	0.976	0.996	0.989

Number of women scoring in each category			
	Percentiles	WHODAS‐36 as “gold standard”	
		P>90	P≤90
WHODAS‐12 as test	P>90	10	2
	P≤90	2	113
	Total	12	115
		P>95	P≤95
WHODAS‐12 as test	P>95	5	1
	P≤95	1	120
	Total	6	121
		P>97.5	P≤97.5
WHODAS‐12 as test	P>97.5	2	1
	P≤97.5	1	123
	Total	3	124

Abbreviations: AUC, area under the curve; P, percentile; ROC, receiver operating characteristic.

**Figure 3:**
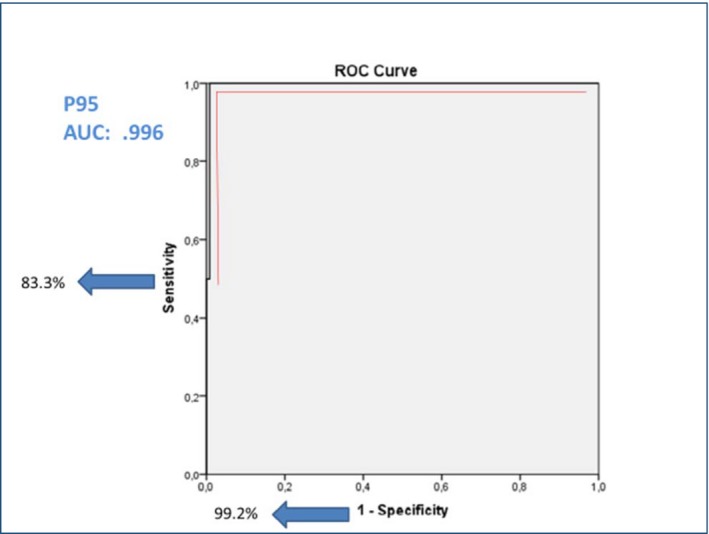
Receiver operating characteristic curve for diagnostic validity of percentile 95 of WHODAS‐12 compared with WHODAS‐36 as “gold standard” to determine dysfunction and disability among women in the postpartum period. Abbreviations: AUC, area under the curve; P, percentile. [Colour figure can be viewed at http://www.wileyonlinelibrary.com

We tested three possible cut‐off points that could have clinical relevance in evaluating WHODAS scores: percentiles 90, 95, and 97.5 were considered (Table 4). The best cut‐off point to diagnose significantly reduced function among postpartum women without any morbidity or complication was the 95th percentile, with the largest area under the curve of 0.996 (Fig. 3).

## 4 DISCUSSION

Given that, as a result of successful efforts to decrease maternal mortality, more women than ever are now surviving childbirth, and that well‐being is understood as a broad spectrum, it is relevant to understand women's functioning during pregnancy and the postpartum period, and the impact of morbidities in causing reduced function. It is important to document standard values of WHODAS scores, in women of reproductive age and in the postpartum period, in order to study the impact of pregnancy and related maternal morbidity on functioning and disability. The present study gives the total and domain scores for WHODAS‐36 in a population with no morbidity at all, compared with scores from women on a continuum of severity (non‐SMM and SMM). To the best of our knowledge, this is the first study to establish baseline WHODAS scores among postpartum women without morbidity.

The WHODAS 2.0 tool was developed to assess difficulties due to health conditions, without distinguishing whether the disability is mental or physical in origin^21^; rather it presents a correlation between injury and disability. When WHODAS 2.0 is applied to postpartum women without morbidity, instead of an injury, the intended correlation is between the complex factors present in the woman's life at the time (involving both physical and emotional changes due to pregnancy, childbirth, and childcare, characterized by concerns about self‐confidence, stress over physical attractiveness, mental and social vulnerabilities) and their effects on her everyday functioning.^22^

Although WHODAS does not measure well‐being,^23^ the scores are strongly influenced by this concept, which itself depends on society, culture, and an individual's references. For instance, in Shanghai, China, a recently published study shows that maternal satisfaction and postpartum well‐being were correlated with giving birth to male infants, and the preference for male children has been well described in Chinese culture.^24^ Cultural diversity determines conceptual diversity of well‐being and this may influence WHODAS score as well.

The increasing trend of WHODAS‐36 total score mean value across the three groups analyzed (without morbidity, non‐SMM, and SMM) may reflect the impact of complications on the new mother. Our analysis suggests that regardless of type, complications influence a woman's perception of her functionality, which correlates with the magnitude of the complication. Otherwise, the differences between the mean scores across the three groups would be larger. Recognizing this is important for establishing the care pathways destined for these women during and after the adverse outcomes, especially for the group classified as having non‐SMM.

Postpartum period functionality is the result of a sum of factors reflected by the WHODAS score. The six domains analyzed reflect general areas of life^16^ and the mean values found in our study may reveal the complexity of the postpartum period and conditions that sometimes go unnoticed by the woman and/or her clinician. The two domains that contributed the most to WHODAS‐36 total score mean value (11.58) among women without medical complications were domains 1 (cognition) and 4 (getting along with people). Domain 1 includes questions about memory, learning new tasks, and problem solving in daily life, factors that are correlated to the woman's need to recognize herself within a new family context with a new role (that of mother), in addition to experiencing the bodily changes associated with pregnancy and the expectation of having to deal with the unknown. Domain 4 questions include difficulties related to sexual activities during the last 30 days. The majority of new mothers resume intercourse within the first 3 months after an uncomplicated pregnancy and the vast majority of women experience at least one problem related to sexual function in the first year after delivery.^25^

On the other hand, when comparing across the three groups, Domain 4 scores remained constant. This is in agreement with the findings of Andreucci et al.,^12^ who applied the Female Sexual Function Index to a Brazilian cohort of women with and without SMM, and found similar mean scores in the population studied.^12^ These issues may be inherent to the pregnancy and postpartum period regardless of the presence or absence of any morbidity.

The WHODAS 12‐item version also provides a brief and valuable measure of disability among postpartum women. Even though the 95th percentile of total mean scores had the largest area under the curve, we hypothesize that the best value for applying WHODAS‐12 as a screening test for diagnosing disability is the 90th percentile, given that the lowest specificity value is required for screening tests and this would allow identification of more women as having some kind of functional impairment, regardless of diagnosis (no morbidity, non‐SMM or SMM). Considering that low‐ and middle‐income countries have limited resources, the WHODAS‐12 item version could be a useful tool, initially for epidemiologic and clinical research, and potentially for becoming a routine screening test for functioning and disability in pregnancy and the postpartum period.

At a time when global efforts to improve maternal health have been prioritized, a baseline WHODAS score among postpartum women without any morbidity is helpful to understand the burden of pregnancy and morbidities overall. It could also help guide strategies to improve antenatal and postpartum care, as well as other studies focusing on specific postpartum conditions.

The increasing WHODAS‐36 mean scores when comparing a population of postpartum women with no morbidity with those on a continuum of severity may reflect the impact of morbidity on postpartum functioning. The diagnostic validity of the WHODAS‐12 means that this brief tool could provide a quick and valuable measurement of impaired functioning among postpartum women. The best cut‐off point to diagnose impaired functioning was the 95th percentile, with the largest area under the curve.

## AUTHOR CONTRIBUTIONS

JM, RTS, MLC, MAP, RCP, ECF, CS, JPG, and JGC had the original idea and developed the analysis plan for the current study. JM, JGC, and MLC wrote the first draft and led the manuscript writing process. MHS conducted the data analysis. LS, DC, VF, MB, KB, and PvD gave important suggestions for the plan of analysis. All authors participated in the editing process, providing essential direction and advice for finalizing the manuscript. LS conceptualized the maternal morbidity measurement initiative. All authors read and approved the final manuscript.

## ADDITIONAL COMMAG AND MMWG GROUP MEMBERS

**COMMAG members:** Carla B. Andreucci, Carina R. Angelini, Juliana P. Ferraz, Dulce M. Zanardi, Rodrigo S. Camargo. **MMWG members:** Sara Cottler, Olubukola Fawole, Tabassum Firoz, Luis Gadama, Atf Ghérissi, Gill Gyte, Michelle Hindin, Anoma Jayathilaka, Amanda Kalamar, Yacouba Kone, Isabelle Lange, Laura A. Magee, Arvind Mathur, Affette McCaw‐Binns, Mark Morgan, Stephen Munjanja, Gathari N. Gichuhi, Max Petzold, Elizabeth Sullivan, Frank Taulo, Özge Tunçalp, Rachel Vanderkruik.

## CONFLICTS OF INTEREST

The authors have no conflicts of interest.
